# AAV8-mediated sVEGFR2 and sVEGFR3 gene therapy combined with chemotherapy reduces the growth and microvasculature of human ovarian cancer and prolongs the survival in mice

**DOI:** 10.3389/fmed.2022.1018208

**Published:** 2022-12-08

**Authors:** Anni Kujala, Elina Valkonen, Hanna Sallinen, Laura Tuppurainen, Hanne Laakso, Elias Ylä-Herttuala, Timo Liimatainen, Jouni Kujala, Otto Jokelainen, Reijo Sironen, Maarit Anttila, Seppo Ylä-Herttuala

**Affiliations:** ^1^Department of Biotechnology and Molecular Medicine, A.I. Virtanen Institute for Molecular Sciences, University of Eastern Finland, Kuopio, Finland; ^2^Department of Gynecology, Kuopio University Hospital, Kuopio, Finland; ^3^School of Medicine, Gynecology, Institute of Clinical Medicine, University of Eastern Finland, Kuopio, Finland; ^4^A.I. Virtanen Institute for Molecular Sciences, University of Eastern Finland, Kuopio, Finland; ^5^Clinical Imaging Center, Kuopio University Hospital, Kuopio, Finland; ^6^Research Unit of Medical Imaging, Physics and Technology, University of Oulu, Oulu, Finland; ^7^Department of Diagnostic Radiology, Oulu University Hospital, Oulu, Finland; ^8^Institute of Clinical Medicine, Pathology and Forensic Medicine, University of Eastern Finland, Kuopio, Finland; ^9^Department of Clinical Pathology, Kuopio University Hospital, Kuopio, Finland; ^10^Heart Center and Gene Therapy Unit, Kuopio University Hospital, Kuopio, Finland

**Keywords:** angiogenesis, lymphangiogenesis, antiangiogenic, MRI, xenograft, ovarian cancer

## Abstract

**Background:**

Vascular endothelial growth factors (VEGFs) are major regulators of intratumoral angiogenesis in ovarian cancer (OVCA). Overexpression of VEGFs is associated with increased tumor growth and metastatic tendency and VEGF-targeting therapies are thus considered as potential treatments for OVCA. Here, we examined the antiangiogenic and antitumoral effects on OVCA of adeno-associated virus 8 (AAV8)-mediated expression of soluble VEGF receptors (sVEGFRs) sVEGFR2 and sVEGFR3 together with paclitaxel and carboplatin chemotherapy.

**Materials and methods:**

Immunodeficient mice were inoculated with human OVCA cell line SKOV-3m. Development of tumors was confirmed with magnetic resonance imaging (MRI) and mice were treated with gene therapy and paclitaxel and carboplatin chemotherapy. The study groups included (I) non-treated control group, (II) blank control vector AAV8-CMV, (III) AAV8-CMV with chemotherapy, (IV) AAV8-sVEGFR2, (V) AAV8-sVEGFR3, (VI) AAV8-sVEGFR2 and AAV8-sVEGFR3, and (VII) AAV8-sVEGFR2 and AAV8-sVEGFR3 with chemotherapy. Antiangiogenic and antitumoral effects were evaluated with immunohistochemical stainings and serial MRI.

**Results:**

Reduced intratumoral angiogenesis was observed in all antiangiogenic gene therapy groups. The combined use of AAV8-sVEGFR2 and AAV8-sVEGFR3 with chemotherapy suppressed ascites fluid formation and tumor growth, thus improving the overall survival of mice. Antitumoral effect was mainly caused by AAV8-sVEGFR2 while the benefits of AAV8-sVEGFR3 and chemotherapy were less prominent.

**Conclusion:**

Combined use of the AAV8-sVEGFR2 and AAV8-sVEGFR3 with chemotherapy reduces intratumoral angiogenesis and tumor growth in OVCA mouse model. Results provide preclinical proof-of-concept for the use of soluble decoy VEGFRs and especially the AAV8-sVEGFR2 in the treatment of OVCA.

## Introduction

Ovarian cancer (OVCA) is the third most common gynecological cancer worldwide with more than 300,000 new cases diagnosed in 2020 (1). OVCA is typically asymptomatic in its early phases, and approximately 80% of OVCA patients are diagnosed with advanced stage disease (2). Although advances in the surgical and chemotherapeutic treatments have improved the prognosis of OVCA, advanced OVCA is still associated with a poor prognosis and the highest mortality among gynecological cancers (3).

Angiogenesis, defined as the formation of new vessels from pre-existing vasculature, has been recognized as an essential part of the solid tumor growth and metastasis of OVCA and other cancer diseases. Angiogenesis is mainly regulated by vascular endothelial growth factors (VEGFs) VEGF-A, VEGF-B, VEGF-C, and VEGF-D, and their respective VEGF-tyrosine-kinase receptors (VEGFRs) VEGFR1, VEGFR2, and VEGFR3. VEGF-A, VEGF-C, and VEGF-D are the ligands of VEGFR2 and regulate angiogenesis, vasculogenesis and vascular permeability, whereas binding of VEGF-C and VEGF-D to VEGFR3 controls lymphangiogenesis and plays a key role in tumor metastasis *via* lymphatic vessels (4, 5). Our previous studies have demonstrated that the intratumoral angiogenesis and tumor growth of OVCA can be suppressed with adenovirus-mediated expression of soluble VEGFR (sVEGFR) decoys that bind VEGFs and inhibit VEGF/VEGFR signaling pathway in a mouse model (6–9). However, adenoviral vectors provide a relatively short-term transgene expression which highlights the need for vectors with a more stable transgene expression, such as adeno-associated virus (AAV) vectors (10).

Debulking surgery and combined use of carboplatin and paclitaxel are current gold standards for the treatment of advanced OVCA. Although most patients will respond well to the treatment, majority of the patients will eventually develop chemoresistance and a relapsed disease (11). Combined use of carboplatin-paclitaxel and antiangiogenic gene therapy could provide a synergistic antitumoral effect and improve the efficacy of current treatment options (12).

The aim of this study was to examine whether the AAV8-mediated sVEGFR2 and sVEFGR3 gene therapy provides a more effective inhibition of intratumoral angiogenesis than sVEFGR2 or sVEGFR3 alone, and if chemotherapy can further improve the antitumoral effect of gene therapy. Our results show that the AAV8-mediated sVEGFR2 and sVEGFR3 gene therapy reduces intratumoral angiogenesis and tumor growth, thus demonstrating the therapeutic potential of antiangiogenic gene therapy in OVCA.

## Materials and methods

### Cell culture

Human-originated OVCA cell line called SKOV-3m adapted to the mouse studies by Sallinen et al. (13) was used in this study. The cell line has been designed to closely resemble the aggressive epithelial phenotype of human OVCA. Cells were cultured in McCoy’s 5A medium (M8403, Sigma-Aldrich, Zwijndrecht, NL) with fetal bovine serum (FBS; Sigma-Aldrich, Zwijndrecht, NL) and 1% penicillin-streptomycin in Opti^®^-MEM (Sigma-Aldrich, Zwijndrecht, NL). Cells were harvested with trypsin, then centrifuged and suspensed in Opti-MEM^®^-GlutaMAXTM (GIBCOTM Life Technologies, USA).

### Viral vectors

AAV8-vectors with a cytomegalovirus (CMV) promoter encoding for sVEGFR2 (AAV8-sVEGFR2) and sVEGFR3 (AAV8-sVEGFR3) were used in this study. AAV8-vector with a CMV promoter without any insert (AAV8-CMV) was used as a blank control. Virus titer in the gene transfers was 1 × 10^11^ vg/mL per virus per mouse, in final volume of 200 μL in 0.9% NaCl. Viral vectors were screened to be free from replication-competent viruses, lipopolysaccharides, and bacteriological contaminants.

### Animal model

Immune-deficient female Balb/cA-nu mice (*n* = 65, Taconic Biosciences Inc.) were used in this study as described by Sallinen et al. (13). All animal works were conducted according to the animal experimentation license approved by the National Animal Experiment Board of the Regional State Administrative Agency of Southern Finland (ESAVI) and carried out following the guidelines of the Finnish Act on Animal Experimentation (62/2006).

Mice were isolated in a pathogen-free environment in the Lab Animal Centre at the University of Eastern Finland. The animal feed, water and bedding were autoclaved. Animals were housed in cages in groups of maximum of five animals, and received water and fodder *ad libitum*. SKOV-3m cells (7.5 × 10^6^ cells) were inoculated with a 22 G needle into the peritoneal cavity to produce intraperitoneal tumors. The animals were anesthetized using isoflurane-oxygen-anesthesia (Isoflurane, Baxter Medical AB) for intravenous (*i.v*.) gene therapy and blood sampling. The anesthesia was initiated with 450 mL air and 4.0–4.5% isoflurane; after the animal had fallen asleep the state of anesthesia was lowered to the maintenance dose of 250 mL air and 1.5–2.0% isoflurane. Gene transfers were performed intravenously to the tail vein 7 days after the SKOV-3m -cell injection.

The animals were carefully followed daily, and their survival monitored. The animals were sacrificed using carbon dioxide euthanasia followed by cervical dislocation when significant changes were observed in their general wellbeing (e.g., > 20% weight loss) or the diameter of the tumors exceeded 1.5 cm. The humane endpoints were established *a priori*. At the sacrifice, all visible tumors, small (ca. 0.5 cm^3^) tissue samples (heart, liver, spleen, kidneys, lungs, intestine, and peritoneum) were collected for histological analyses. All tumor tissues were weighed and the amount of ascites fluid was measured.

### Study groups

Mice were randomly assigned into three control groups and four antiangiogenic gene therapy receiving treatment groups (Table 1). Control groups included (I) non-treated group, (II) blank control group with AAV8-CMV, and (III) group with combined AAV8-CMV and chemotherapy. Treatment groups received (IV) AAV8-sVEGFR2, (V) AAV8-sVEGFR3, (VI) combined AAV8-sVEGFR2 and AAV8-sVEGFR3, and (VII) combined AAV8-sVEGFR2, AAV8-sVEGFR3, and chemotherapy.

**TABLE 1:** Study groups.

Study group	*N*	Vector (s)	Titer vg/mL	Chemotherapy
I (non-treated control)	11	–	–	–
II (blank control)	10	AAV8-CMV	1 × 10^11^	–
III	9	AAV8-CMV	1 × 10^11^	Paclitaxel (20 mg/kg) + carboplatin (80 mg/kg)
IV	9	AAV8-sVEGFR2	1 × 10^11^	–
V	7	AAV8-sVEGFR3	1 × 10^11^	–
VI	10	AAV8-sVEGFR2 AAV8-sVEGFR3	1 × 10^11^ 1 × 10^11^	–
VII	9	AAV8-sVEGFR2 AAV8-sVEGFR3	1 × 10^11^ 1 × 10^11^	Paclitaxel (20 mg/kg) + carboplatin (80 mg/kg)

All animals received 7.5 × 10^6^ SKOV-3m cells injected intraperitoneally. Animals in the treatment groups received either AAV8-sVEGFR2, AAV8-sVEGFR3, or AAV8-sVEGFR2 and AAV8-sVEGFR3 gene transfers, coupled with chemotherapy.

### Magnetic resonance imaging

Magnetic resonance imaging (MRI) was used to evaluate the development of the intraperitoneal tumors, ascites fluid formation and the efficiency of the gene therapy. MRI was first performed 6 days after the SKOV-3m cell injection, and then weekly until the death of the animals (days 10, 17, 24, 31, and 38 from the gene transfer). In the MRI measurements, a horizontal 7 T magnet was utilized (Bruker PharmaScan, Bruker BioSpin MRI GmbH, Germany) and interfaced with ParaVision 5.1, using a quadrature volume transmitter and a 3 cm diameter loop surface receiver coil. The animals were anesthetized initially with 4% isoflurane (Baxter Oy, Helsinki, Finland) in 70% N_2_–30% O_2_, and during imaging, the isoflurane level was reduced to approximately 1.5%. During the MRI, the animal’s respiration rate, which was used to trigger the imaging, was monitored with a pneumatic pillow (SA Instruments, Stony Brook, NY, USA). Physiological temperature of the mice was maintained by a warm water pad.

Tumors were identified from the surrounding tissues by their location as well as their anomalous intensity and contrast. Anatomical imaging was done using multislice T2 -weighted fast spin echo sequence [repetition time (TR) = 3 s, effective echo time (TE) = 44 ms, echo train length = 4, 2 averages, field of view (FOV) = 40 × 40 mm^2^, matrix size = 256 × 256, slice thickness 1 mm, 30 slices] with images covering the abdominal cavity of the animals. From the anatomical MRI images, the size of the tumors was analyzed and followed weekly until the sacrification of the animals. The area of the tumor was hand drawn on anatomical images and the total tumor volumes from multiple tumor loci were then calculated.

From the T2-weighted anatomical images, one slice showing the largest tumor in the top part of the mice abdomen was selected for the diffusion weighted imaging. The apparent diffusion coefficient (ADC) of water was determined by diffusion weighted imaging using a spin echo sequence with diffusion weighting in three orthogonal directions (TR = 2 s, TE = 27 ms, *b*-value = 500 s/mm^2^, diffusion time = 14 ms; gradient duration = 4 ms; FOV = 40 × 40 mm^2^, matrix size = 128 × 64). In addition, one image without diffusion weighting (*b* = 0) was collected. ADC maps were calculated on a pixel-by-pixel basis using equation


$$A\,D\,C=-\frac{1}{b}\,\text{In}\,\left(\frac{S_{D\,W\,I}}{S_{0}}\right)$$

where *b* is the *b*-value, *S*_*DWI*_ is the signal from trace diffusion weighted image, and *S*_0_ is the signal from b = 0 image. The Aedes software package (aedes.uef.fi) and Matlab (MathWorks, Natick, MA) were used for MRI analysis. All MRI analyses were done blindly.

### Chemotherapy

Combined chemotherapy of carboplatin and paclitaxel was given 7 days after the gene transfer to study groups III and VII. Intraperitoneally administered doses for carboplatin (10 mg/mL, Hospira, Hospira Uk Limited) and paclitaxel (Hospira, 6 mg/mL; Hospira UK Limited) were 80 and 20 mg/kg per mouse.

### Histology and immunohistochemistry

Collected tissue samples were immersed overnight in 4% paraformaldehyde in phosphate-buffered saline (PBS), followed by overnight immersion in 15% sucrose. Tissue samples were processed to 5 μm thick paraffin embedded sections and used for histological and immunohistological analyses.

Presence of treatment-induced liver and tumor necrosis was blindly evaluated from haematoxylin-eosin (HE) (Sigma-Aldrich, Zwijndrecht, NL) stained sections by pathologist. Stained histological sections were photographed with Nikon DS-Ri2 confocal microscope and processed with NIS-Elements AR 4.30.02 (Nikon, Tokyo, Japan), ImageJ 1.48v (National Institutes of Health, USA) and Photoshop CS (Adobe, California, USA) softwares.

Antiangiogenic effect of treatment was evaluated from CD34-A488 –fluorescent immunostained tumor sections. The paraffin-embedded tumor slices were mounted on glass slides. The samples were deparaffinized and hydrated, then washed with Triton X-100 solution (1:500 dilution in PBS) before citrate buffer (10 mM, pH 6.0) boiling. The samples were then blocked with serum (1:10 diluted goat serum and 1:50 diluted mouse serum) at room temperature, before being incubated with monoclonal primary CD34 antibody (1:50 dilution, MEC 14.7, HM1015, Hycult Biotech, Uden, the Netherlands) overnight in 4°C in humid chamber. The samples were then incubated with the secondary antibody (1:50 dilution, Alexa Fluor™ 488 Goat Anti-Rat IgG (H + L), Invitrogen by Thermo Fisher Scientific, Waltham, Massachusetts, USA) at room temperature, and after that with Sudan Black B (0.1% SBB in 70% ethanol). The samples were washed with Tween20 (0.02% Tween in PBS) before being enclosed in mounting medium (Vectashield with DAPI, H-1200, Vector) and covered with a microscope slide. The stained sections were photographed with Nikon DS-Qi2 confocal microscope and stained blood vessels were manually measured from 10 representative fields (100 × magnification) with NIS-Elements AR 4.30.02. Mean vascular density (MVD) and total vascular area (TVA) were calculated from acquired results.

Cell proliferation was estimated with KI67 immunohistological staining. For the KI67 staining, the paraffin-embedded tumor slices were mounted on glass slides. The samples were deparaffinized and hydrated, then boiled in citrate buffer (pH 6.0), and treated with hydrogen peroxide (5%). The samples were then blocked with serum (1.5% normal horse serum in PBS) at room temperature, before being incubated with monoclonal primary KI67 antibody (1:50 dilution, clone MIB-1 Dako M7240, Dako-Cytomation, Glostrup, Denmark) overnight in 4°C in humid chamber. The samples were then incubated with the secondary antibody (1:100 dilution, biotinylated Horse Anti-Mouse IgG (H + L), Vector laboratories Inc, Newark, CA, USA) at room temperature. Then a 45-min Avidin-biotin-HRP step was performed using the Vectastain Elite ABC -kit (Vector laboratories Inc, Newark, CA, USA), after which the samples were covered with tetrahydrochloride 3,3′- diaminobenzidine (DAB) at room temperature, counterstained with Harris haematoxylin, dehydrated, enclosed in Permount mounting medium (Thermo Fisher Scientific, Waltham, Massachusetts, USA), and covered with a microscope slide. The stained sections were photographed with Nikon DS-Ri2 confocal microscope and the proportion of KI67 positive cells was calculated from 10 representative fields (100 × magnification) with NIS-Elements AR 4.30.02 software.

### Statistical analyses

Statistical analyses were performed using IBM SPSS Statistics 27 software (IBM, Armonk NY, USA). Study groups were compared with one-way ANOVA followed by LSD *post-hoc* analysis or Kruskal-Wallis one-way analysis of variance when appropriate. Log_10_ transformation was used for KI67 results. Survival data was analyzed with Kaplan-Meier Estimate and Kruskal-Wallis followed by Logrank test. Fixed effects assay based on linear mixed model was used to analyze longitudinal MRI results. All results are shown as mean ± standard error of mean (SEM). Results were considered statistically significant with values of *p* < 0.05*, *p* < 0.01^**^, and *p* < 0.001^***^.

## Results

### Microvessel measurements

**FIGURE 1:**
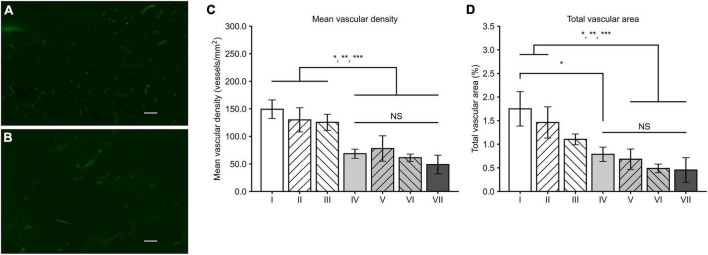
Tumor CD34 stainings and analysis of MVD and TVA. CD34-positive microvessels in the groups II **(A)** and VII **(B)** with a magnification of 100 × and scale bar of 100 μm. Density of microvessels is visibly higher in a tumor from the group II. **(C)** In general, MVD was lower in all the groups receiving sVEGFR2 and/or sVEGFR3 gene therapy (groups IV–VII) when compared to the groups I–III. **(D)** Similar to MVD results, TVA was generally lower in the groups IV–VII while difference to the group III was less prominent. Results were considered statistically significant with values of *p* < 0.05*, *p* < 0.01**, and *p* < 0.001***.

MVD and TVA measured from CD34-positive microvessels (Figure 1) revealed that in general, MVD was lower in all treatment groups (groups IV-VII) when compared to the control groups (groups I-III). The lowest MVD was observed in the group VII (48.9 ± 16.9 / mm^2^). However, the differences between gene therapy receiving groups remained non-significant. The MVD was significantly lower in the group VII when compared to the groups I (*p* = 0.001), II (*p* = 0.008), and III (*p* = 0.013). TVA data follows the same trend as with the MVD data: the lowest TVA was found in group VII (0.45 ± 0.26%), in comparison to the other groups. The TVA was statistically significantly lower in group VII compared to the groups I (*p* = 0.006) and II (*p* = 0.032).

### Histology

**FIGURE 2:**
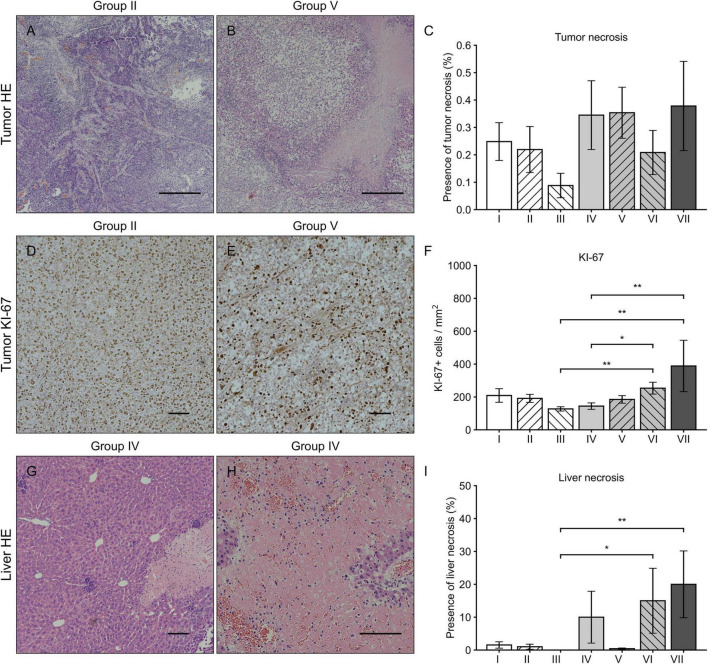
HE and Ki-67 immunohistochemical stainings of tumor and liver. Tumor HE stainings from the group II **(A)** and VII **(B)** with magnification 40 × and scale bar of 500 μm. Varying levels of tumor necrosis were observed in all groups without significant differences **(C)**. Tumor Ki67 stainings from the group II **(D)** and VII **(E)** with magnification 100 × and scale bar of 100 μm. The number of Ki67 positive cells was highest in the groups VI and VII when compared to the groups III and IV **(F)**. Liver HE stainings from the group IV with magnification 100 × **(G)** and 200 × **(H)** with a scale bar of 100 μm. In general, the presence of liver necrosis was higher in all groups receiving sVEGFR2 as a part of the therapy (groups IV, VI, and VII) of which the groups VI and VII showed significant differences when compared to the group III **(I)**. Results were considered statistically significant with values of *p* < 0.05* and *p* < 0.01**.

Histologically the tumors were high-grade epithelial ovarian carcinomas. No significant difference was observed in tumor necrosis between the groups (*p* = 0.237, Kruskal-Wallis). At the end of the follow-up, the morphology of the OVCA tissue had been completely or partially replaced by necrotic tissue in all groups, some groups displaying only small local necrosis (Figures 2A–C). Cell proliferation index measured by KI67 immunohistochemical staining was the highest in the group VII (389 ± 156 / mm^2^) when compared to the other groups (Figures 2D–F). Significant differences in liver necrosis (Figures 2G–I) were observed between the groups (*p* = 0.001, Kruskal-Wallis): pairwise comparison showed that liver necrosis was more prominent in the groups VI and VII when compared to group III (*p* = 0.030 and *p* = 0.009, Kruskal-Wallis).

### Formation of ascites fluid

**FIGURE 3:**
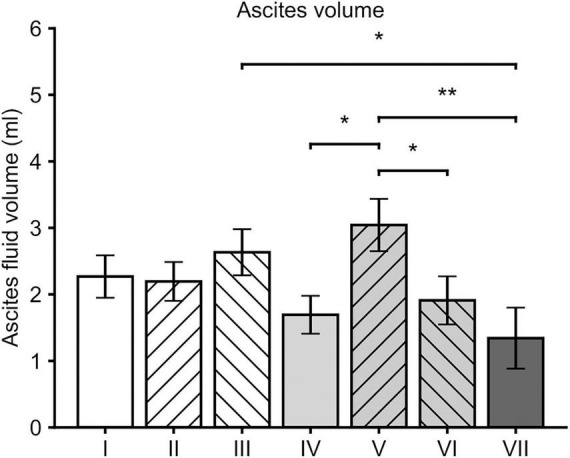
Mean ascites volumes at the time of sacrification. In general, all the groups that received sVEGFR2 as a part of the gene therapy (groups IV, VI and VII) showed lower volumes of ascites when compared to the group V. When compared to the group III, group VII was the only group with a significantly lower ascites volume. Results were considered statistically significant with values of *p* < 0.05* and *p* < 0.01**.

Mean volume of ascites fluid in all groups was 2.16 ± 0.14 mL. Ascites fluid formation was significantly reduced in group VII when compared to the groups III (*p* = 0.017, *Post-hoc* LSD) and V (*p* = 0.003). Significantly reduced amounts of ascites fluid were detected also in the groups IV (*p* = 0.015) and VI (*p* = 0.031) when compared to group V (Figure 3).

### Magnetic resonance imaging

**FIGURE 4:**
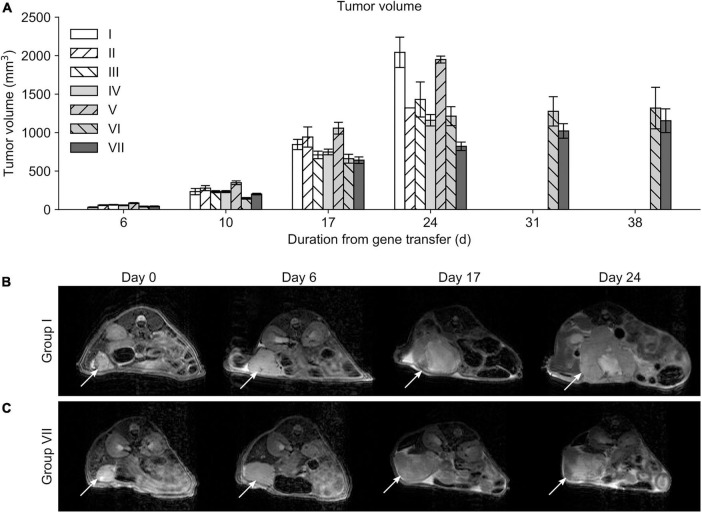
Mean tumor volumes. **(A)** Differences in tumor volumes were first observed at day 10 only between the groups V and VI. Only mice belonging to the study groups VI and VII survived long enough to be measured at timepoints 31 and 38 days. Serial MRI scans from the group I **(B)** and VII **(C)** demonstrating tumor growth during the follow-up. Location of the tumor is indicated with a white arrow.

At day 10 the tumor volumes of mice in the group V were significantly larger compared to mice in the group VI (*p* = 0.018) (Figure 4A). Most of the significant differences in the tumor volumes between the groups were detected at day 17 and 24 MRI scans; at day 17 group VII showed significantly smaller tumor volumes compared to groups I (*p* = 0.015), II (*p* < 0.001), and V (*p* < 0.001) (Figures 4B,C). At day 24 MRI scans, the smallest tumor volumes were detected in the group VII when compared to other groups (*p* < 0.001). After this timepoint MRI data could only be acquired from animals from groups VI and VII, because mice from all other groups had to be sacrificed due to sacrification criteria. Group VII showed significantly reduced tumor volumes compared to group VI at day 31 (*p* = 0.003) and at day 38 (*p* = 0.041).

In general, mice in group VII had higher ADC values at day 6 when compared to control groups (group I, *p* < 0.001; group II, *p* = 0.022; group III, *p* = 0.029), or group IV (*p* = 0.001) while differences with mice in group V were less prominent. ADC values of mice in group VII declined over time and no significant differences in ADC values were observed after 24 days follow-up. ADC values between all study groups are presented in Supplementary Figure 1.

### Survival

**FIGURE 5:**
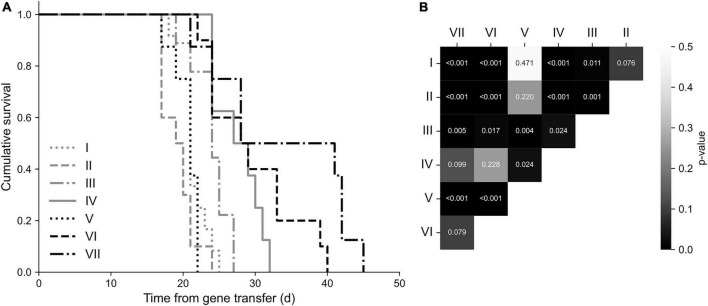
Survival analysis. **(A)** Kaplan-Meier survival plot. The longest overall survival was observed in the group VII. **(B)** Statistically significant differences between the groups presented on a correlogram. Animals in the group VII had significantly prolonged survival time (*p* < 0.001) compared to the groups I, II, V, and III (*p* = 0.005).

The overall mean survival time of mice was 25 ± 1 days from SKOV-3m cell injections. Mice in group VII had the longest overall survival, 34 ± 3 days. There were statistically significant differences between the study groups (*p* < 0.001, Kruskal-Wallis): group VII had significantly prolonged survival when compared to the control groups and group V (Figure 5). Also, group III had significantly prolonged survival time compared to groups I (*p* = 0.011) and II (*p* = 0.001).

## Discussion

VEGF/VEGFR targeting antiangiogenic therapies are an attractive approach to treat OVCA due to the angiogenesis-dependent growth of the tumor and a less likely development of resistance (14). Increasing number of VEGF/VEGFR targeting therapeutic agents have been introduced during the past few years, but only a few agents—such as bevacizumab, pazopanib, and nintedanib—have significantly improved survival in clinical trials (11). Gene therapy can provide a more sustained expression of antiangiogenic factors and therefore help to achieve a prolonged treatment effect. Our approach targets the ligands of both VEGFR2 and VEGFR3, which should provide a more comprehensive inhibition of angiogenesis and lymphangiogenesis and improve the therapeutic effect as compared to other agents, such as bevacizumab, which block only one of the members of the VEGF family. To our knowledge, no preclinical AAV8-mediated gene therapy trials to combine both sVEGFR2 and sVEGFR3 in OVCA mouse models have been reported.

We showed that AAV8-sVEGFR2, AAV8-sVEGFR3, and combined AAV8-sVEGFR2 and AAV8-sVEGFR3 gene therapies all reduce MVD and the TVA in the tumor tissue. Our previous studies have provided similar results by demonstrating that the combined use of adenovirus-mediated sVEGFR1 (AdsVEGFR1), AdsVEGFR2, and AdsVEGFR3 gene therapy (6, 7) and the combination of AdsVEGFR2 and AdsVEGFR3 gene therapy (8) all reduce MVD and TVA in the same mouse model. Unlike Sallinen et al., we observed that sVEGFR2 and sVEGFR3 alone are able to suppress tumor angiogenesis in a manner similar to combined sVEGFR2 and sVEGFR3 gene therapy. What has remained open is the benefit of combining the sVEGFR2 and sVEGFR3, and using antiangiogenic gene therapy with chemotherapy. Chemotherapy is associated with several adverse effects that impair the quality of life, such as nausea, fatigue and neurotoxicity (15). It is tempting to think that the combined use of gene therapy and chemotherapy could allow us to decrease the dose of chemotherapy without losing the antitumoral effect, thus decreasing the incidence of chemotherapy-induced adverse effects. However, this requires further studies to investigate the achieved treatment response with different gene therapy and chemotherapy doses.

Similar to our recent study (9), decreased intratumoral angiogenesis was not directly reflected by the KI67 expression. Mice receiving the combined AAV8-sVEGFR2 and AAV8-sVGEFR3 treatment showed signs of upregulated KI67 expression, thus suggesting that gene therapy promoted proliferation of tumor cells. It has been hypothesized that the promoted proliferation of cancer cells might result from compensatory mechanisms that are activated as a result of VEGF/VEGFR inhibition (16). The presence of compensatory mechanisms that support cell survival and viability during antiangiogenic treatment may partly explain why the therapy does not completely suppress the tumor growth.

The combined use of AAV8-sVEGFR2 and AAV8-sVEGFR3 with chemotherapy was found to decrease intraperitoneal ascites formation when compared to the chemotherapy group. The reduction in ascites fluid formation appears to be associated with AAV8-sVEGFR2, while AAV8-sVEGFR3’s effect seems to be less prominent. It must be noted that ascites volumes in the control groups were lower than expected, possibly due to the aggressive growth of tumors which resulted in sacrification before the ascites fluid had accumulated in the abdominal cavity. The link between VEGF/VEGFR signaling and ascites fluid formation still requires further investigation, but it has been suggested that upregulated VEGF expression increases vascular permeability and promotes ascites formation in OVCA (17). Since the formation of ascites fluid is associated with a poor prognosis and severely impaired quality of life (18), the reduction in ascites fluid formation is an important treatment effect.

Serial MRI imaging supports the antitumoral effect of AAV8-mediated sVEGFR2 gene therapy. Our results show that both the AAV8-sVEGFR2 and chemotherapy restricted tumor growth while AAV8-sVEGFR3 alone did not significantly restrict tumor growth. Likewise, mice receiving coupled AAV8-sVEGFR2, AAV8-sVEGFR3, and chemotherapy showed the most restricted tumor growth, thus demonstrating possible synergistic benefits from the combined use of antiangiogenic treatment and chemotherapy. Previous studies (6, 7) observed a similar antitumoral effect with the combined use of AdsVEGFR1, AdsVEGFR2, and AdsVEGFR3. In contrast to our results, Sallinen et al. did not observe a notable antitumoral effect with AdsVEGFR2 or AdsVEGFR3 alone. We observed a temporary ADC increase in mice that received combined AAV8-sVEGFR2 and AAV8-sVEGFR3 gene therapy, which might indicate a positive treatment response (19). However, far-reaching conclusions should not be drawn from the ADC results.

Survival analysis supported the observed antiangiogenic and antitumoral effects. The overall survival of the treated mice was improved in all the groups that received AAV8-sVEGFR2 gene therapy as a part of the treatment. Although the longest median overall survival times were observed in mice that received the combined AAV8-sVEGFR2, AAV8-sVEGFR3 and chemotherapy, the achieved improvement in the survival did not significantly differ from the groups that received AAV8-sVEGFR2, or coupled AAV8-sVEGFR2 and AAV8-sVEGFR3 without chemotherapy.

AAV8 has a high affinity for hepatocytes and mild liver toxicity is a relatively common adverse effect of AAV8-mediated gene transfer (20). We observed varying levels of liver toxicity in all AAV8-sVEGFR2 treated groups while AAV8-sVEGFR3 and chemotherapy did not significantly induce liver toxicity, thus suggesting that especially AAV8-sVEGFR2 is associated with mild liver toxicity. In general, AAV vectors are considered to be well tolerated and highly efficient viral vectors, and studies with longitudinal follow-ups have not shown significant adverse effects with commonly used viral doses (21). Nevertheless, the observed toxicity should be treated with caution and further toxicological studies on the topic are well warranted.

## Conclusion

Our results show that the combined use of AAV8-sVEGFR2 and AAV8-sVEGFR3 with chemotherapy significantly restricts intratumoral angiogenesis and tumor growth in murine xenograft model of human OVCA, thus demonstrating the potential synergistic benefits of coupled antiangiogenic therapy and chemotherapy. Although both the AAV8-sVEGFR2 and AAV8-sVEGFR3 were found to suppress intratumoral angiogenesis, antitumoral effects of the treatment were mostly driven by AAV8-sVEGFR2, which efficiently reduced intratumoral angiogenesis, ascites formation and tumor growth, and improved the overall survival of treated mice. Our results provide proof-of-concept of the use of soluble decoy VEGFRs and especially the AAV8-sVEGFR2 in the treatment of OVCA with clear potential for further preclinical and clinical development.

## Data availability statement

The raw data supporting the conclusions of this article will be made available by the authors, without undue reservation.

## Ethics statement

The animal study was reviewed and approved by the National Animal Experiment Board of the Regional State Administrative Agency of Southern Finland (ESAVI).

## Author contributions

AK and EV performed the animal work, MRI imaging, conducted laboratory and computer analyses on the samples, and wrote the first draft of the manuscript. LT, HS, MA, TL, and SY-H contributed to conception and design of the study. AK performed the statistical analysis. OJ and RS gave a clinical pathologist’s opinion on sample histology. EY-H and HL consulted about MRI and contributed to the MRI imaging. LT, HL, and JK wrote sections of the manuscript. All authors contributed to manuscript revision, read, and approved the submitted version.
