# Supplementary material for: AAV8-mediated sVEGFR2 and sVEGFR3 gene therapy combined with chemotherapy reduces the growth and microvasculature of human ovarian cancer and prolongs the survival in mice
Source: Front Med (Lausanne). 2022 Dec 8;9:1018208. doi: 10.3389/fmed.2022.1018208 (PMC9773272; doi:10.3389/fmed.2022.1018208)
Supplement: Supplementary file 1 [file Image_1.pdf]

Supplementary Material

1 Supplementary Figures

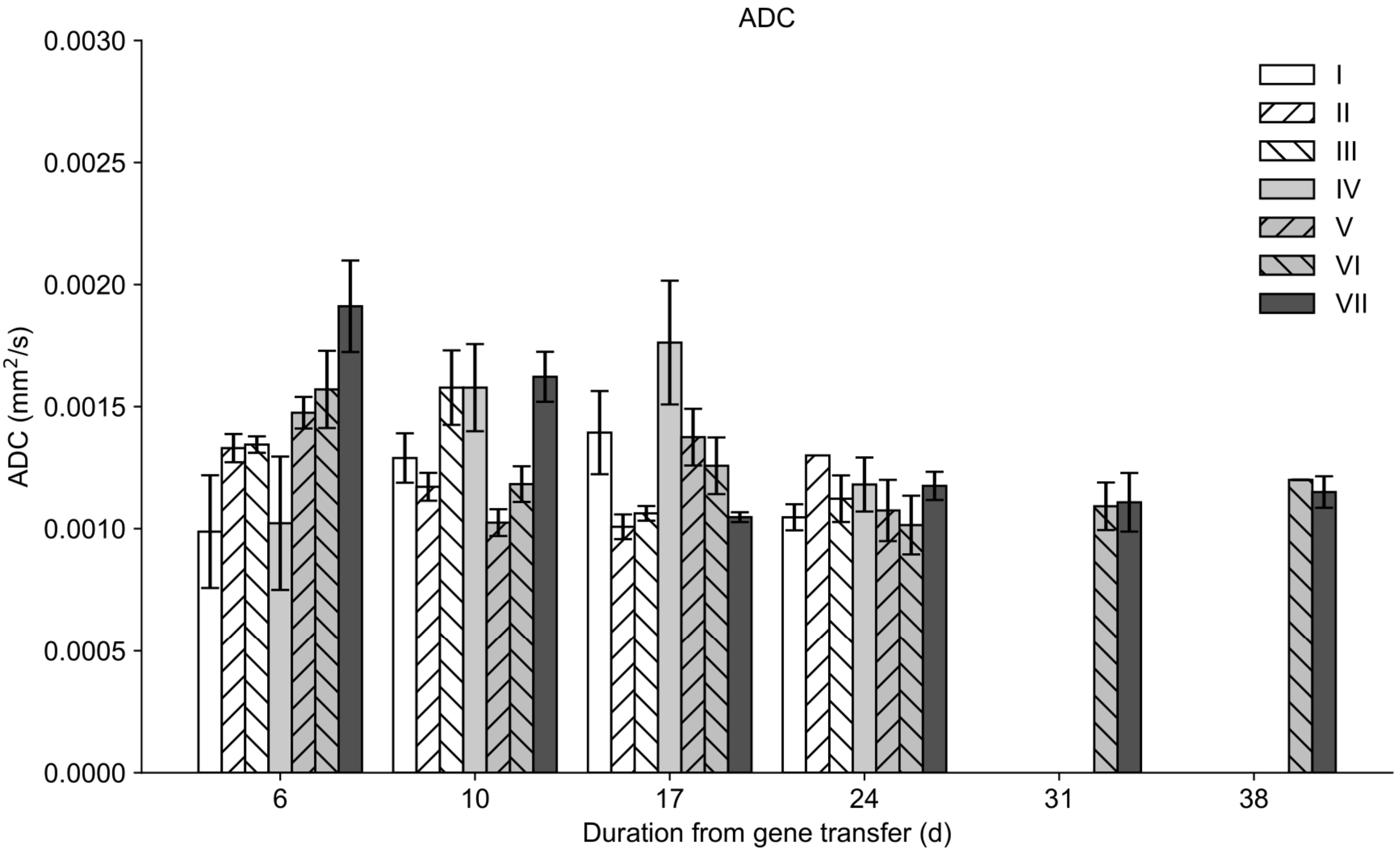

Supplementary Figure 1. Means of ADC values of study groups over time.
